# Generalized cell phenotyping for spatial proteomics with language-informed vision models

**DOI:** 10.1101/2024.11.02.621624

**Published:** 2024-11-17

**Authors:** Xuefei (Julie) Wang, Rohit Dilip, Yuval Bussi, Caitlin Brown, Elora Pradhan, Yashvardhan Jain, Kevin Yu, Shenyi Li, Martin Abt, Katy Börner, Leeat Keren, Yisong Yue, Ross Barnowski, David Van Valen

**Affiliations:** 1 Division of Biology and Biological Engineering, Caltech, Pasadena, CA; 2 Division of Computing and Mathematical Science, Caltech, Pasadena, CA; 3 Department of Intelligent Systems Engineering, Luddy School of Informatics, Computing, and Engineering, Indiana University, Bloomington, IN; 4 Department of Mathematics and Computer Science, Weizmann Institute of Science, Rehovot, Israel; 5 Department of Molecular Cell Biology, Weizmann Institute of Science, Rehovot, Israel; 6 Howard Hughes Medical Institute, Chevy Chase, MD

## Abstract

We present a novel approach to cell phenotyping for spatial proteomics that addresses the challenge of generalization across diverse datasets with varying marker panels. Our approach utilizes a transformer with channel-wise attention to create a language-informed vision model; this model’s semantic understanding of the underlying marker panel enables it to learn from and adapt to heterogeneous datasets. Leveraging a curated, diverse dataset with cell type labels spanning the literature and the NIH Human BioMolecular Atlas Program (HuBMAP) consortium, our model demonstrates robust performance across various cell types, tissues, and imaging modalities. Comprehensive benchmarking shows superior accuracy and generalizability of our method compared to existing methods. This work significantly advances automated spatial proteomics analysis, offering a generalizable and scalable solution for cell phenotyping that meets the demands of multiplexed imaging data.

## 1 Introduction

Understanding the structural and functional relationships present in tissues is a challenge at the forefront of basic and translational research. Recent advances in multiplexed imaging have expanded the number of transcripts and proteins that can be quantified simultaneously^1–10^, opening new avenues for large-scale analysis of human tissue samples. Concurrently, advances in deep learning have shown immense potential in integrating information from both image and natural language to build foundation models, and these approaches have also shown to be promising for various biomedical imaging applications^11,12^. However, a critical question persists: how can these innovative methods be harnessed to transform the vast amounts of data generated by multiplexed imaging into meaningful biological insights?

This paper proposes a novel language-informed vision model to solve the problem of generalized cell phenotyping in spatial proteomic data. While the new data generated by modern spatial proteomic platforms are exciting, significant challenges in analyzing and interpreting these datasets at scale remain. Unlike flow cytometry or single-cell RNA sequencing, tissue imaging is performed with intact specimens. Thus, to extract single-cell data, individual cells must be identified - a task known as cell segmentation - and the resulting cells must be examined to determine their cell type and which markers they express - a task known as cell phenotyping. A general solution for cell phenotyping has proven more challenging for several reasons. First, it requires scalable, automated, and accurate cell segmentation, which has only recently become available^13–17^. Second, imaging artifacts, including staining noise, marker spillover, and cellular projections, pose a formidable challenge to phenotyping algorithms^18–20^. Third, general phenotyping algorithms must handle the substantial differences in marker panels, cell types, and tissue architectures across experiments. Each new dataset often has a different number of markers, each with its own distinct meaning. Existing approaches to meet this challenge range from conventional methods that require manual gating and clustering^21–23^ to more recent machine learning-based solutions^18–20,24–26^. While representing significant breakthroughs, these tools have failed to scale for two reasons. First, many of them rely on human intervention, which places a fundamental limit on their ability to scale to big data. Second, these methods cannot handle the wide variability in marker panels that exist across experiments. Some methods require labeling and re-training for new datasets; even when transfer learning is possible^18–20^, the target dataset must share similarities in the marker panel with the source dataset. Bridging this gap requires a versatile model that can be trained on multiple datasets and performance inference on new data with unseen markers.

To this end, we developed an end-to-end cell phenotyping model capable of learning from and generalizing to diverse datasets, regardless of their specific marker panels. Our approach was twofold. First, we began by curating and integrating a large, diverse set of spatial proteomics data that includes a substantial amount of publicly available datasets in addition to all the datasets generated by the NIH HuBMAP consortium to date. Human experts generated labels with a human-in-the-loop framework for representative fields of view (FOV) for each dataset member; we term the resulting labeled dataset Expanded TissueNet. Second, we developed a new deep learning method, DeepCellTypes, that is capable of learning how to perform cell phenotyping on these diverse data. This architecture incorporates language and vision encoders, enabling it to leverage information from raw marker images and the semantic information associated with language describing different markers and cell types. We employed a transformer architecture with the channel-wise attention mechanism to integrate this visual and linguistic information, thereby eliminating the dependence on specific marker panels. When trained on Expanded TissueNet, our method demonstrates state-of-the-art accuracy across a wide spectrum of cell types, tissue types, and spatial proteomics platforms. Moreover, we demonstrate that DeepCellTypes has superior zero-shot cell phenotyping performance compared to existing methods and can generalize to new datasets with unseen markers. Both Expanded TissueNet and DeepCellTypes are made available through the DeepCell software library with permissive open-source licensing.

## 2 Results

Here, we describe three key aspects of our work - constructing Expanded TissueNet, the deep learning architecture of DeepCellTypes, and our model training strategy designed to improve generalization.

### 2.1 DeepCell Label enables scalable construction of ExpandedTissueNet

**Figure 1:**
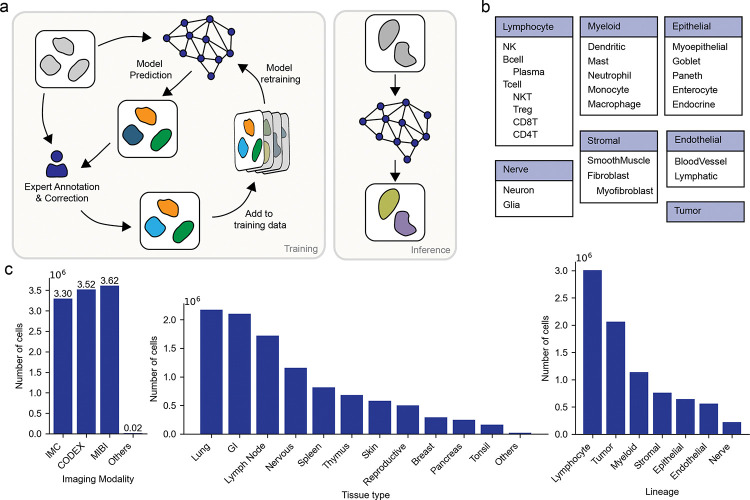
Construction of Expanded TissueNet with Expert-in-the-loop labeling. a) We constructed Expanded TissueNet by integrating human experts into a human-in-the-loop framework. Cell phenotype labels are generated by having an expert either label fields of view from scratch or by correcting model errors. We adapted our image labeling software DeepCell Label to facilitate visualization, inspection, and phenotype labeling of spatial proteomic datasets. Newly labeled data were added to the training dataset to enable continuous model improvement. b) Expanded TissueNet covers 7 major cell lineages, each containing a hierarchy of cell types. The labels for these 28 specific cell types enable the training of our cell phenotype model. c) Expanded TissueNet contains over 10 million cells; here, we show the number of labeled cells across 6 imaging platforms, 13 tissues, and 7 lineages.

Training data quality, diversity, and scale are at the foundation of robust and generalizable deep learning models. To create a dataset that captured the diversity of marker panels, cellular morphologies, tissue heterogeneity, and technical artifacts present in the field, we first compiled data from published sources^19,27–40^, as well as unpublished data deposited in the HuBMAP data portal. For each dataset, we collected raw images, corresponding channel names, and cell type labels (when available). Each dataset was resized to a standard resolution of 0.5 microns per pixel. A key step in our process was standardizing marker names and cell types across all datasets to enable cross-dataset comparisons, integration, and analysis. Cell types were organized by lineage as shown in Fig. 1b. We then performed whole-cell segmentation with Mesmer^13^ and mapped any existing cell type labels to the resulting cell masks. When quality labels did not exist, we generated them through a human-in-the-loop labeling framework that leveraged expert labelers. Marker positivity labels were generated by manually gating the mean signal intensity for each marker for each dataset. To accelerate this step, we extended DeepCell Label, our cloud-based software for distributed image labeling, to the cell type labeling task (Fig. S3). DeepCell Label allows users to visualize images, analyze marker intensities, refine segmentation masks, and annotate cell types.

The resulting dataset, Expanded TissueNet, consists of 10.5 million cells, spanning six imaging platforms: Imaging Mass Cytometry (IMC)^3^, CO-Detection by indEXing (CODEX)^2^, Multiplex Ion Beam Imaging (MIBI)^1^, Iterative Bleaching Extends Multiplexity (IBEX)^5^, MICS (MACSima Imaging Cyclic Staining)^4^, and Multiplexed immunofluorescence (MxIF) with Cell DIVE^*TM*^ technology (Leica Microsystems, Wetzlar, Germany), with the majority of data coming from the first three. The dataset covers 13 common tissue types and 28 specific cell types across 7 broad cell lineages, providing a diverse representation of human biology. Across all datasets, we cataloged 177 unique protein markers with an average of 27 markers per dataset.

To generate single-cell images to train cell phenotyping models, we extracted a 64×64 patch from each marker image centered on each cell to capture an image of that cell and its surrounding neighborhood. These images were augmented with two binary masks: a self-mask delineating the central cell (1’s for pixels inside the cell and 0’s for pixels outside) and a neighbor-mask capturing surrounding cells within the patch. This approach preserves cell morphology, marker information, and spatial context for model training.

### 2.2 DeepCellTypes is a language-informed vision model with a channel-wise transformer

**Figure 2:**
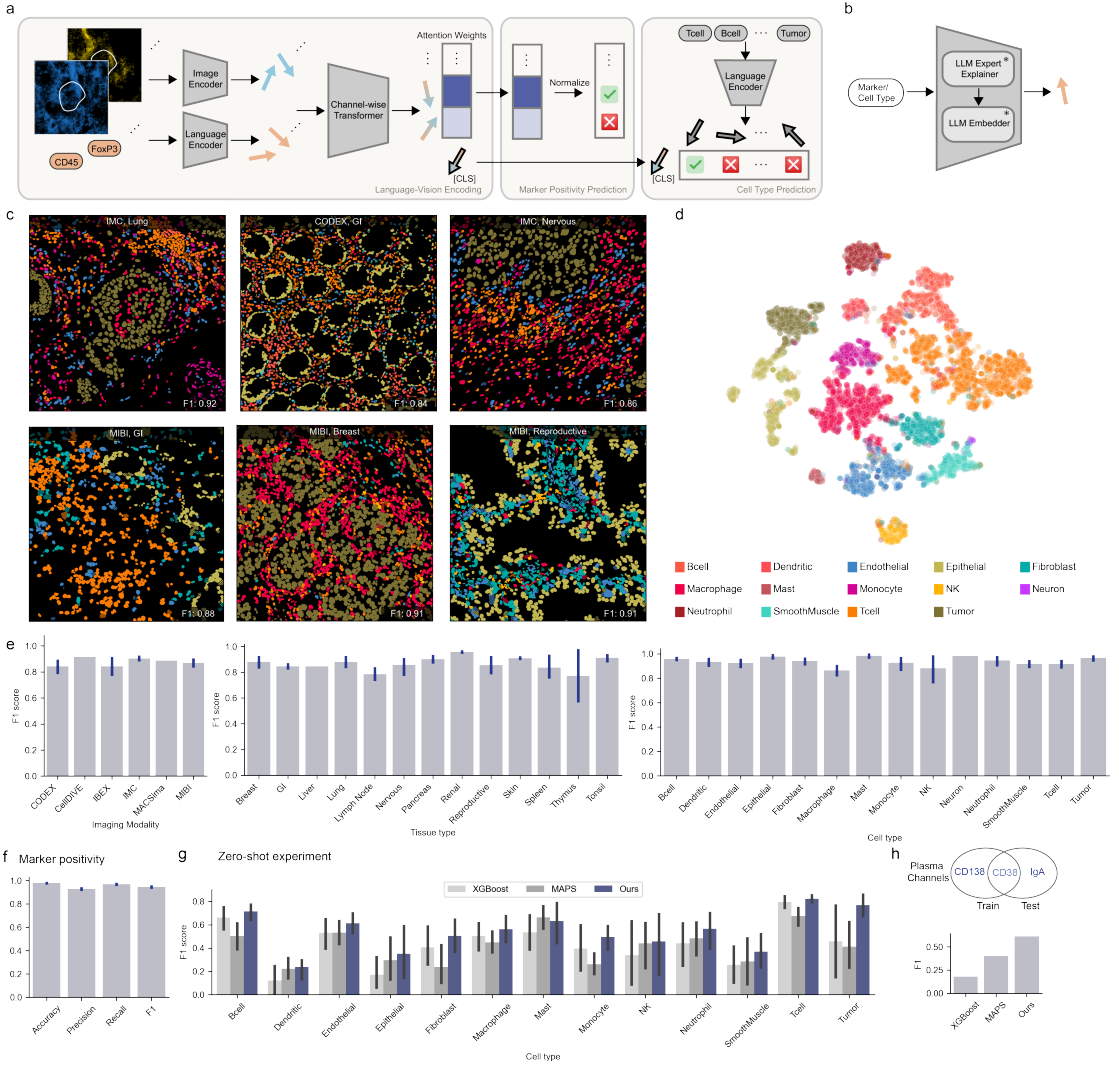
DeepCell Types enables generalized cell phenotyping. a) Model Design: Image patches and marker names are processed by image and language encoders to produce image embeddings (blue arrows) and text embeddings (orange arrows), respectively. A channel-wise transformer module combines these embeddings, generating marker (blue-orange blended arrows) and cell representations ([CLS] arrow). Attention weights predict marker positivity, while the [CLS] token’s embedding is used for contrastive cell type prediction, enabling flexible processing of varied marker panels. b) Language Encoder: An LLM expert explainer retrieves relevant knowledge by prompting an LLM to generate detailed descriptions of queried markers or cell types. An LLM embedder then converts these descriptions into vector representations. c) Example Field of Views (FOVs) with cell types colorized, error predictions are masked by crossed lines. d) Latent Space Visualization: Cell types form distinct clusters, demonstrating our model’s ability to learn biologically relevant features independent of imaging modalities or dataset origins. e) Classification performance analyzed by imaging modalities, tissue types and cell types. DeepCell Type’s model architecture and diverse dataset facilitate generalization. f) Marker positivity performance. g-h) Comparison of zero-shot generalization performance against baselines. Models were evaluated by holding out a single dataset before training and evaluating performance on the held-out dataset. Each dataset was held out once; we report the average performance across all models. DeepCellTypes outperforms existing methods and can generalize well even when alternative markers are used to identify a cell type.

We developed DeepCellTypes, a cell phenotyping model with the unique ability to learn from and adapt to diverse datasets with different marker panels. Our model consists of three main components: a visual encoder, a language encoder, and a channel-wise transformer (Fig. 2a).

#### Visual Encoder:

The visual encoder processes 64×64 cell patches from each channel, along with corresponding self-masks and neighbor-masks. This module employs a Convolutional Neural Network (CNN) to condense the image patches into embedding vectors, capturing spatial information about staining patterns, cell morphology, marker expression levels, and neighborhood context.

#### Language Encoder:

To incorporate semantic understanding of markers and cell types, we employed a language encoder that uses a large language model (LLM) to generate semantically rich embedding vectors in a two-step process (Fig. 2b): First, a frozen LLM explainer is prompted to extract comprehensive knowledge about a marker or cell type, capturing general information, marker-cell type relationships, and alternative names. Next, a frozen LLM embedder converts this semantic information into an information-rich embedding vector. This approach leverages knowledge about the biology of markers and cell types, enabling our model to understand the meaning behind each channel.

#### Channel-wise Transformer:

To allow our model to generalize across marker panels, we use a transformer module with channel-wise attention. This module adds a marker’s image embedding with its corresponding language embedding and applies self-attention to the combined representation. Crucially, this self-attention operation is applied across all the markers, similar to how a human might look across the marker images to interpret a stain. While transformers for sequence data typically include a positional encoding, we did not include one. This design choice preserved the length-and-order invariance of self-attention^41^ and allowed our model to process inputs from diverse marker panels without modification. We appended a learnable [CLS] token to represent the overall cell and to aggregate information across all channels. The normalized attention weights between this [CLS] token and the marker embeddings in the final layer provide interpretable marker positivity scores. This architecture serves as a fusion point for visual and linguistic information, enabling our model to discern cross-channel correlations while also understanding the biological significance of each marker.

### 2.3 Improved cross-platform generalization through contrastive and adversarial learning

Our training strategy extends the joint vision-language approach from the model architecture to loss function design. Instead of relying on a standard classification loss, we employ the Contrastive Language-Image Pretraining (CLIP) loss, which has demonstrated remarkable efficacy in integrating image and text information across various tasks^42^. We use the same language encoder to embed markers and cell types. During training, our language-informed vision model was tasked with aligning each cell’s image-marker embedding (e.g., the cell’s [CLS] token embedding taken from the final transformer layer) with the embedding of its corresponding cell type name (Fig. 2a). It was also tasked with minimizing the similarity between the cell’s image-marker embedding with embeddings of incorrect cell type names. This contrastive training approach unifies the visual ([CLS] token embeddings) and textual (cell-type-name embeddings) representations in a shared latent space, enhancing the model’s ability to capture nuanced semantic meanings between different cell types. We used a focal-enhanced^43^ of the CLIP loss to deal with class imbalance by assigning higher importance to difficult examples. Furthermore, we applied a binary cross entropy loss to the normalized attention weights to force alignment between the attention weights and marker positivity. For this loss, we used label smoothing^44,45^ to prevent the model from becoming over-confident.

During the development of Expanded TissueNet, we noted significant class imbalances with respect to imaging modalities. To prevent the model from learning representations that are overfit to any single modality, we implemented an auxiliary task that encourages the model to learn invariant representations across imaging platforms. To do so, we added a classification head that takes [CLS] token embeddings and predicts imaging modalities and coupled it with a gradient reversal layer^46^. During backpropagation, the gradients were reversed, thus teaching the model to ‘unlearn’ modality-specific differences. We found the resulting model developed more robust and general features and focused on the underlying biological characteristics of cells rather than platform-specific features (Fig. 2d, Fig. S2c).

### 2.4 Benchmarking

We sought to evaluate our model’s performance across diverse datasets against other state-of-art approaches. Our analysis encompassed various imaging modalities, tissue types, and cell types, providing a comprehensive view of the model’s effectiveness. As illustrated in Fig. 2e, our model demonstrates robust performance across all these dimensions. Notably, the model’s performance remained strong even for underrepresented imaging modalities like MACSima, highlighting its ability to generalize beyond the dominant data sources. Fig. 2c showcases example images, visually demonstrating the model’s efficacy across diverse inputs. Additionally, the model also performs well in marker positivity prediction, as evidenced in Fig. 2f.

To assess the modality-invariance of learned features, we applied a two-step dimensionality reduction technique to the cell embeddings: Neighborhood Components Analysis (NCA)^47^ followed by t-SNE^48^. This approach, previously shown to be effective in revealing latent space structure without overfitting^49^, allowed us to visualize the organization of our model’s latent space. The resulting visualization, presented in Fig. 2d, reveals that the embedding space is primarily organized by cell types rather than imaging modalities (Fig. S2c). This organization demonstrates the model’s focus on biologically relevant features over platform-specific characteristics.

We conducted a series of hold-out experiments to evaluate our model’s zero-shot generalization capabilities. In each experiment, we excluded one dataset from training, trained the model on the remaining data, and then tested its performance on the held-out dataset. We benchmarked our model against two baselines: XGBoost^50^, a tree-boosting algorithm, and MAPS^20^, a neural network-based approach. We note that in this evaluation, differences in marker panels are effectively flagged as missing data for XGBoost and set to zero for MAPS. As shown in Fig. 2g, our model demonstrates favorable performance compared to the baselines across most cell types. This demonstrates that our language-informed vision model effectively leverages semantic understanding to achieve superior generalization across marker panels compared to previous approaches. Moreover, it underscores the utility of integrating language in vision information to meet the challenges of heterogeneous spatial proteomics data.

Another key advantage of our language-informed approach is the model’s ability to generalize to unseen markers. We demonstrated this capability using the GVHD dataset^40^, where plasma cells are identified using a unique marker (IgA) instead of the more common CD138 marker. Despite sharing only the CD38 marker with other datasets (which is not exclusive to plasma cells), our model robustly recognizes plasma cells in the GVHD dataset when trained on all other datasets (Fig. 2g). This further showcases the model’s ability to understand the semantic information inherent in the names of markers and cell types, enabling effective generalization to new marker panels.

## 3 Discussion

This work addresses a critical challenge in spatial proteomics: the need for automated, generalizable, and scalable cell phenotyping tools. Our approach directly tackled the fundamental issue of variability in marker panels across different experiments, enabling learning from multiple data sources. Trained on a diverse dataset, our model demonstrated robust and accurate performance across various experimental conditions, outperforming existing methods in zero-shot generalization tasks.

Our model’s use of raw image data as input represents a significant advancement over traditional cell phenotyping approaches originated from non-spatial single-cell technologies like scRNA-Seq and flow cytometry. These methods rely on mean intensity values extracted from segmented cells and often fall short in coping with the artifacts of spatial proteomic data. Our work adds to the growing body of evidence that image-centric approaches are more robust to segmentation errors, noises, and signal spillovers^18,26^.

A key innovation in our model is the integration of language components to enhance generalization. By incorporating textual information about cell types and markers, we leverage broader biological knowledge that extends beyond the training data. The synergy between visual and linguistic information allows our language-informed vision model to integrate information across a broad set of experimental data, achieving superior performance compared to traditional machine-learning approaches. A key advantage of our approach is its ability to train on multiple datasets simultaneously, leveraging marker correlations across datasets, a capability lacking in existing methods. This unified framework allows continuous model improvement as new data emerges, consolidating the field’s knowledge into a single, increasingly comprehensive model. Given the success of language-informed vision models in handling the heterogeneous marker panels here, applying this methodology to image-based spatial transcriptomics or marker-aware cell segmentation would be a natural extension of this work.

Despite these advances, our method has limitations that merit discussion. First, our zero-shot experiment demonstrates a common characteristic of machine learning systems: while the performance is optimal within the domain of training data, it inevitably degrades when encountering samples too far out-of-domain. Despite our best efforts, Expanded TissueNet does not exhaust all imaging modalities and tissue types. Hence, even though we showed promising improvement compared to existing methods, datasets that substantially diverge from ExpandedTissueNet may require additional labeling and model finetuning to achieve adequate performance. We anticipate our labeling software and human-in-the-loop approaches to labeling will enable these efforts and facilitate the collection and labeling of increasingly diverse data, further improving model performance. As demonstrated here and previously^13^, integrated data and model development is viable for consortium-scale deep learning in the life sciences. Second, while our work enables generalization across marker panels, generalization across cell types remains an open challenge, given the wide array of tissue-specific cell types. Addressing this limitation is a crucial direction for future investigation. Last, our model is trained on cell patches and cannot access the full image. While constraining the model’s effective receptive field can help generalization, it can limit accuracy as features at longer length scales like functional tissue units and anatomical structures can provide context to aid cell type determination. Multi-scale language-informed vision models that integrate local and global features may offer a novel avenue for enhanced performance.

In conclusion, DeepCellTypes marks a significant advancement in spatial proteomics data analysis. By directly addressing the challenge of generalization across marker panels, we enabled accurate cell-type labeling for spatial proteomics data generated throughout the cellular imaging community and laid the groundwork for future innovations in cellular image analysis.

## 8 Methods

### 8.1 Data preprocessing and standardization

We implemented several preprocessing steps that were applied to each marker image independently to ensure consistent and high-quality input for our model. All images were resized to a resolution of 0.5 microns per pixel (mpp). We normalized the images by scaling pixel values based on the 99th percentile of all non-zero values across FOVs for each channel and each dataset. The resulting images were subsequently clipped between 0 and 5 to mitigate the impact of extremely bright pixels. Processed images were saved in zarr format, enabling chunked, compressed storage and fast loading. We also standardized marker names and cell type labels across datasets, identifying and combining alternative names to ensure consistency.

Cell segmentation was performed using the Mesmer algorithm^13^. We identified nuclear channels (e.g., DAPI, Histone H3) and cytoplasm/membrane channels (e.g., Pan-Keratin, CD45) to generate accurate whole-cell masks. For each segmented cell, we extracted a 64×64 pixel patch centered on the cell. For cells near image boundaries, we added padding to ensure complete 64×64 patches. To capture segmentation information, we appended a self-mask and a neighbor-mask to each channel’s raw image. This resulted in images of shape (*C,* 3*,* 64*,* 64), where *C* is the number of marker images varying by dataset. We zero-padded the tensor to *C*_*max*_ = 87 to allow processing by regular neural networks. A binary padding mask of (*C*_*max*_*,*) is also generated for later use in the transformer module. Marker positivity labels were generated by manually gating the mean signal intensity for each marker for each dataset.

### 8.2 Model architecture

Our model employs a hybrid architecture, combining Convolutional Neural Network (CNN) and Transformer components to effectively process both image and textual data. Our model architecture has four components:

- *Image encoder*: The image encoder consists of an 11-layer CNN with 2D convolution layers, followed by SiLU activation and batch normalization. We reshape the input tensors from (*B, C*_*max*_*,*3*,*64*,*64) to (*B* ∗ *C*_*max*_*,* 3*,* 64*,* 64), allowing a single CNN to process all channels regardless of their marker correspondence. This design ensures the model remains agnostic to specific marker representations. The CNN output is then reshaped into embeddings of size (*B, C*_*max*_*,* 256).
- *Text encoder*: For the text encoder, we utilized OpenAI’s GPT-4 model^51^ as the expert explainer and the GPT-3^52^ text-embedding-3-large model as the embedder. We prompt the explainer to provide detailed descriptions of each marker type including its general description, alternative names, and cell type correspondence. The resulting 1024-dimensional embeddings are linearly mapped to 256 dimensions to match the image embeddings.
- *Channel-wise transformer*: To merge information contained in the image and text embeddings, we fed them into a transformer module that uses channel-wise attention. A learnable [CLS] token was appended to the embedding tensor to represent the entire cell. The transformer module comprises 5 encoder layers with a hidden dimension of 256 and a feed-forward dimension of 512. We employ a padding mask to exclude dummy channels, ensuring the model focuses only on real channels. This flexible mechanism accommodates varying numbers of input channels.
- *Gradient reversal*: We employed a gradient reversal technique to mitigate systematic bias induced by different imaging modalities. The [CLS] token is fed into a 3-layer MLP classification head for predicting imaging modalities. During backpropagation, we reverse the gradient of the first layer. This approach minimizes the loss on cell type classification while maximizing the loss on imaging modality classification, effectively removing modality-specific information from the learned representations.

To determine marker positivity, the attention weights between the [CLS] token and all other tokens were extracted, normalized to the range [0, 1], and interpreted as marker positivity values. For cell type classification, we first extracted the [CLS] token embedding from the output layer and reused the text encoder to convert cell type names into embeddings. We trained the [CLS] token and cell type name tokens using the contrastive (e.g., CLIP) loss^42^, calculating cosine similarities with a ground truth similarity of 1 for the correct cell type and 0 for all others.

### 8.3 Model training

Our training strategy incorporates three distinct losses: cell type classification, marker positivity prediction, and reverse imaging modality classification. We assign constant weights to the first two components while employing a ramping weight for the third, proportional to the quartic root of the number of epochs. This approach allows the model to initially learn representations best suited for cell type classification and marker positivity prediction before gradually moving towards modality-invariant features to enhance generalization performance.

For cell type classification, we implement a focal version of the contrastive loss^42,43^ with gamma = 2.0. This adaptation addresses class imbalance and improves performance on more challenging categories. The marker positivity prediction utilizes binary cross-entropy loss with label smoothing = 0.2, a technique known to prevent overfitting^44,45^.

**Algorithm 1:** Focal CLIP Contrastive Loss

1	**procedure** FocalCLIPLoss(I,T,γ,τ)	
2	**Input:** Image embeddings I	
3	**Input:** Text embeddings T	
4	**Input:** Focusing parameter γ	
5	**Input:** Learnable scaling parameter τ	
6	$I\leftarrow I/∥I{∥}_{2},T\leftarrow T/∥T{∥}_{2}$	▷ Normalize embeddings
7	$S\leftarrow IT^{⊤}/\tau$	▷ Similarity matrix
8	$P_{\mathrm{img}\;}\leftarrow softmax(S)$	
9	$P_{\mathrm{txt}\;}\leftarrow softmax\left(S^{⊤}\right)$	
10	Y←[0,1,...,N-1]	▷ Groundtruth indices
11	**procedure** FocalLoss(P, Y)	
12	ce←NLLLoss(P,Y)	
13	$P_{t}\leftarrow P[i,Y[i]]$ for all i	▷ Probabilities of all true classes
14	$\mathrm{loss}\;\leftarrow {\left(1-P_{t}\right)}^{\gamma }\cdot ce$	▷ Focal-weighted loss
15	**return** mean(loss)	
16	**end procedure**	
17	$L_{img}\leftarrow \;\mathrm{FocalLoss}\;\left(L_{img}\right)$	
18	$L_{txt}\leftarrow \;\mathrm{FocalLoss}\;\left(L_{txt}\right)$	
19	$L\leftarrow \left(L_{img}+L_{txt}\right)/2$	▷ Symmetric loss
20	**return** L	
21	**end procedure**	

We train the model for 15 epochs using the RAdam optimizer with a learning rate of 10^−4^. We employ several data augmentation techniques to boost generalization, including random flipping, rotation, and image resizing. Further, we randomly drop out 8 marker channels during training to encourage the model to learn robust features that are less dependent on specific markers. We also added random gaussian noises to marker name embeddings and cell type embeddings to prevent overfitting and to improve robustness.

### 8.4 Baselines

To benchmark our model’s performance, we implemented two established approaches: XGBoost^50^ and MAPS^20^. We began by extracting features from each dataset and calculating the mean intensity value for each available marker channel in every cell. Next, we compiled a list of all unique markers present across all datasets, which served as a universal reference for data alignment. We then harmonized the data by aligning each dataset to this universal marker list. For markers present in a dataset, we used the calculated mean intensity values, while for absent markers, we inserted a placeholder value (‘nan’ for XGBoost, 0 for MAPS).

We implemented XGBoost using the Python XGBoost package (https://github.com/dmlc/xgboost). Placeholder values were explicitly indicated through the data matrix interface. We trained the XGBoost model for 200 epochs using default parameters.

For MAPS, we used the implementation available at https://github.com/mahmoodlab/MAPS. In addition to the marker expression data, we appended a column representing cell size to the input matrix, as per the MAPS protocol. The MAPS model was trained using default parameters for 500 epochs.

## Supplementary Material

Supplement 1
