# Generalized cell phenotyping for spatial proteomics with language-informed vision models

**DOI:** 10.1101/2024.11.02.621624

**Published:** 2025-08-22

**Authors:** Xuefei (Julie) Wang, Rohit Dilip, Ahamed Raffey Iqbal, Yuval Bussi, Caitlin Brown, Elora Pradhan, Yashvardhan Jain, Kevin Yu, Shenyi Li, Martin Abt, Katy Börner, Leeat Keren, Yisong Yue, Ross Barnowski, David Van Valen

**Affiliations:** 1 Division of Biology and Biological Engineering, Caltech, Pasadena, CA; 2 Division of Computing and Mathematical Science, Caltech, Pasadena, CA; 3 Department of Intelligent Systems Engineering, Luddy School of Informatics, Computing, and Engineering, Indiana University, Bloomington, IN; 4 Department of Mathematics and Computer Science, Weizmann Institute of Science, Rehovot, Israel; 5 Department of Molecular Cell Biology, Weizmann Institute of Science, Rehovot, Israel; 6 Howard Hughes Medical Institute, Chevy Chase, MD

## Abstract

We present DeepCell Types, a novel approach to cell phenotyping for spatial proteomics that addresses the challenge of generalization across diverse datasets with varying marker panels collected across different platforms. Our approach utilizes a transformer with channel-wise attention to create a language-informed vision model; this model’s semantic understanding of the underlying marker panel enables it to learn from and adapt to heterogeneous datasets. Leveraging a curated, diverse dataset named Expanded TissueNet with cell type labels spanning the literature and the NIH Human BioMolecular Atlas Program (HuBMAP) consortium, our model demonstrates robust performance across various cell types, tissues, and imaging modalities. Comprehensive benchmarking shows superior accuracy and generalizability of our method compared to existing methods. This work significantly advances automated spatial proteomics analysis, offering a generalizable and scalable solution for cell phenotyping that meets the demands of multiplexed imaging data.

## Teaser

A vision model with semantic understanding of markers enables generalizable cell phenotyping for diverse spatial proteomics data.

## 1 Introduction

Understanding the structural and functional relationships present in tissues is a challenge at the forefront of basic and translational research. Recent advances in multiplexed imaging have expanded the number of transcripts and proteins that can be quantified simultaneously (1, 2, 3, 4, 5, 6, 7, 8, 9, 10), opening new avenues for large-scale analysis of human tissue samples. Concurrently, advances in deep learning have shown immense potential in integrating information from both image and natural language to build foundation models, and these approaches have also shown to be promising for various biomedical imaging applications (11, 12). However, a critical question persists: how can these innovative methods be harnessed to transform the vast amounts of data generated by multiplexed imaging into meaningful biological insights?

This paper proposes a novel language-informed vision model to solve the problem of generalized cell phenotyping in spatial proteomic data. While the new data generated by modern spatial proteomic platforms is exciting, significant challenges in analyzing and interpreting these datasets at scale remain. Unlike flow cytometry or single-cell RNA sequencing, tissue imaging is performed with intact specimens. Thus, to extract single-cell data, individual cells must be identified - a task known as cell segmentation - and the resulting cells must be examined to determine their cell type and which markers they express - a task known as cell phenotyping. A general solution for cell phenotyping has proven more challenging for several reasons. First, it requires scalable, automated, and accurate cell segmentation, which has only recently become available (13, 14, 15, 16, 17). Second, imaging artifacts, including staining noise, marker spillover, and cellular projections, pose a formidable challenge to phenotyping algorithms (18, 19, 20). Third, general phenotyping algorithms must handle the substantial differences in marker panels, cell types, and tissue architectures across experiments. Each new dataset often has a different number of markers, each with its own distinct meaning. Existing approaches to meet this challenge range from conventional methods that require manual gating and clustering (21, 22, 23) to more recent machine learning-based solutions (24, 25, 18, 20, 19, 26). QuPath (23) and histoCAT (22) are comprehensive interactive platforms that enable users to analyze cells based on their marker expressions and spatial characteristics, often incorporating lightweight clustering or classification tools. FlowSOM (21) is a commonly used clustering algorithm based on self-organizing maps. Two prominent probabilistic models, Celesta (24) and Astir (25), enable automated cell phenotyping by incorporating prior knowledge of cell type marker mappings, bypassing the need for labeled data. Celesta (24) employs a Markov Random Field with a mean-field approximation, while Astir (25) is built on variational inference with Gaussian Mixture Models. Supervised approaches like MAPS (20) (a feed-forward neural network), Stellar (19) (a graph neural network), and CellSighter (18) (a convolutional neural network), offer the advantage of accurate and specific cell type identification when labeled data is available. Complementing these, Nimbus (26) provides a deep learning model for predicting marker positivity, which can then be used with clustering algorithms like FlowSOM (21) for cell type identification. While these tools highlight the advances the field has made, existing methods face significant challenges in scaling for three reasons. First, many of them rely on human intervention, which places a fundamental limit on their ability to scale to big data. Second, these methods cannot handle the wide variability in marker panels that exist across experiments. Some methods require labeling and re-training for new datasets. Even when transfer learning is possible (18, 19, 20), the target dataset must share significant similarities in the marker panel with the source dataset. Third, most existing methods have been developed for data collected from a single imaging platform. Bridging this gap requires a versatile model that can be trained on multiple datasets and perform inference on new data with unseen markers.

To this end, we developed an end-to-end cell phenotyping model capable of learning from and generalizing to diverse datasets, regardless of their specific marker panels. Our approach was twofold. First, we began by curating and integrating a large, diverse set of spatial proteomics data that includes a substantial amount of publicly available datasets in addition to all the datasets generated by the NIH HuBMAP consortium to date. Human experts generated labels with a human-in-the-loop framework for representative fields of view (FOV) for each dataset member; we term the resulting labeled dataset Expanded TissueNet. Second, we developed a new deep learning method, DeepCell Types, that is capable of learning how to perform cell phenotyping on these diverse data. This architecture incorporates language and vision encoders, enabling it to leverage information from raw marker images and the semantic information associated with language describing different markers and cell types. We employed a transformer architecture with the channel-wise attention mechanism to integrate this visual and linguistic information, thereby eliminating the dependence on specific marker panels. When trained on Expanded TissueNet, our method demonstrates state-of-the-art accuracy across a wide spectrum of cell types, tissue types, and spatial proteomics platforms. Moreover, we demonstrate that DeepCell Types has superior zero-shot cell phenotyping performance compared to existing methods. Both Expanded TissueNet and DeepCell Types are made available through the DeepCell software library with permissive open-source licensing.

## 2 Results

Here, we describe three key aspects of our work - (1) constructing Expanded TissueNet, (2) the deep learning architecture of DeepCell Types, and (3) our model training strategy designed to improve generalization.

### 2.1 DeepCell Label enables scalable construction of Expanded TissueNet

**Figure 1:**
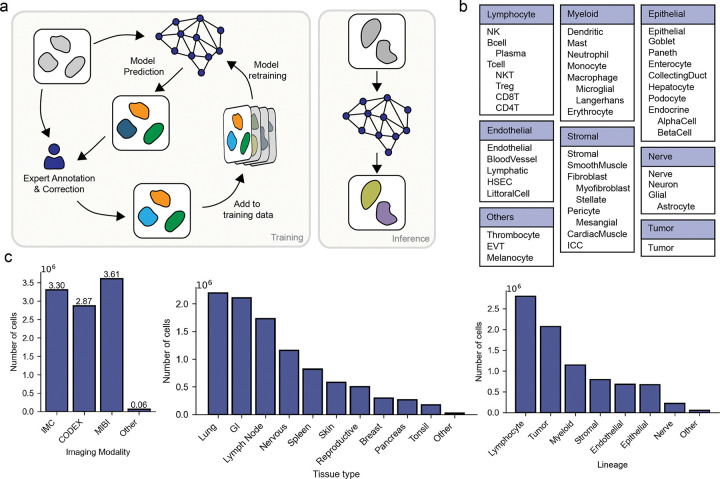
Construction of Expanded TissueNet with Expert-in-the-loop labeling. a) We constructed Expanded TissueNet by integrating human experts into a human-in-the-loop framework. Beyond existing dataset annotations, cell phenotype labels are generated by having an expert either label fields of view from scratch or by correcting model errors. We adapted our image labeling software DeepCell Label (Fig. S3) to facilitate visualization, inspection, and phenotype labeling of spatial proteomic datasets. Newly labeled data were added to the training dataset to enable continuous model improvement. b) Expanded TissueNet covers 8 major cell lineages, each containing a hierarchy of cell types. The labels for these 48 specific cell types enable the training of our cell phenotype model. c) Expanded TissueNet contains over 9.8 million cells; here, we show the number of labeled cells across 9 imaging platforms, 17 tissues, and 8 lineages.

Training data quality, diversity, and scale are at the foundation of robust and generalizable deep learning models. To create a dataset that captured the diversity of marker panels, cellular morphologies, tissue heterogeneity, and technical artifacts present in the field, we first compiled data from published sources (27,28,29,30,31,32,33,34,35,36,37,38,39,19,40,41,42,43), as well as unpublished data deposited in the HuBMAP data portal. For each dataset, we collected raw images, corresponding channel names, and cell type labels (when available). Each dataset was resized to a standard resolution of 0.5 microns per pixel. A key step in our process was standardizing marker names and cell types across all datasets to enable cross-dataset comparisons, integration, and analysis. Cell types were organized by lineage as shown in Fig. 1b. We then performed whole-cell segmentation with Mesmer (13) and mapped any existing cell type labels to the resulting cell masks. When quality labels did not exist, we generated them through a human-in-the-loop labeling framework that leveraged expert labelers (Fig. 1a).To establish marker positivity labels, experts manually gated the mean signal intensity for each marker and cell type within each dataset, leveraging their extensive knowledge of marker-cell type associations. To accelerate this step, we extended DeepCell Label, our cloud-based software for distributed image labeling, to the cell type labeling task (Fig. S3). DeepCell Label allows users to visualize images, analyze marker intensities, refine segmentation masks, and annotate cell types.

The resulting dataset, Expanded TissueNet, consists of 9.8 million cells, spanning nine imaging platforms: Imaging Mass Cytometry (IMC) (3), CO-Detection by indEXing (CODEX) (2), Multiplex Ion Beam Imaging (MIBI) (1), Iterative Bleaching Extends Multiplexity (IBEX) (5), MICS (MACSima Imaging Cyclic Staining) (4), and Multiplexed immunofluorescence with Cell DIVE^*TM*^ technology (Leica Microsystems, Wetzlar, Germany), Cyclic Immunofluorescence (CycIF) (44), InSituPlex (Ultivue), Vectra (Akoya Science), with the majority of data coming from the first three. The dataset covers 17 common tissue types and 48 specific cell types across 8 broad cell lineages, providing a diverse representation of human biology (Fig. 1c). Across all datasets, we cataloged 262 unique protein markers with an average of 24.6 markers per dataset.

To generate single-cell images to train cell phenotyping models, we extracted a 64×64 patch from each marker image centered on each cell to capture an image of that cell and its surrounding neighborhood. These images were augmented with two binary masks: a self-mask delineating the central cell (1’s for pixels inside the cell and 0’s for pixels outside) and a neighbor-mask capturing surrounding cells within the patch. This approach preserves cell morphology, marker information, and spatial context for model training.

### 2.2 DeepCell Types is a language-informed vision model with a channel-wise transformer

**Figure 2:**
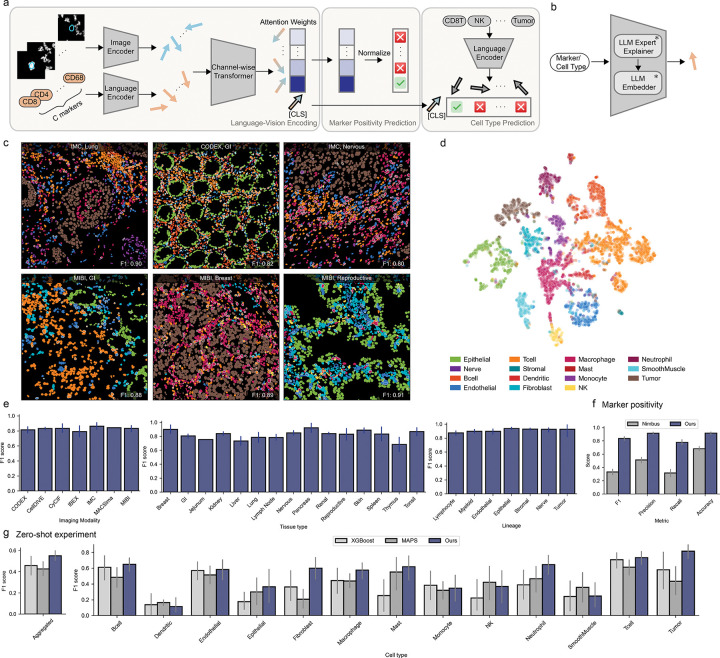
DeepCell Types enables generalized cell phenotyping. a) Model Design: Image patches and marker names are processed by image and language encoders to produce image embeddings (blue arrows) and text embeddings (orange arrows), respectively. A channel-wise transformer module combines these embeddings, generating marker (blue-orange blended arrows) and cell representations ([CLS] arrow). Attention weights predict marker positivity, while the [CLS] token’s embedding is used for contrastive cell type prediction. b) Language Encoder: An LLM expert explainer retrieves relevant knowledge by prompting an LLM to generate detailed descriptions of queried markers or cell types. An LLM embedder then converts these descriptions into vector representations. c) Example Field of Views (FOVs) with cell types colorized, error predictions are masked by crossed lines. d) Latent Space Visualization: Cell types form distinct clusters, demonstrating our model’s ability to learn biologically relevant features independent of imaging modalities or dataset origins. e) Classification performance analyzed by imaging modalities, tissue types, and lineages. To ensure adequate statistical power for this analysis, categories with fewer than 100 samples were excluded. f) Comparison of marker positivity performance between ours and Nimbus on our dataset, the comparison on Nimbus dataset is shown in Fig. S4f. g) Comparison of zero-shot generalization performance against XGBoost and MAPS. Models were evaluated using leave-one-dataset-out cross-validation on 13 large datasets ( 20,000 cells). After consolidating cell types into common categories and excluding rare ones (present in 25% of the experiments or with 500 samples), we reported both aggregated scores (left, XGBoost: 0.458 +/− 0.172, MAPS: 0.427 +/− 0.117, DeepCell Types: 0.551 +/− 0.08) and per-category scores (right).

We developed DeepCell Types, a cell phenotyping model with the unique ability to learn from and adapt to diverse datasets with different marker panels. Our model consists of three main components: a visual encoder, a language encoder, and a channel-wise transformer (Fig. 2a).

#### Visual Encoder:

The visual encoder processes 64×64 cell patches from each channel, along with corresponding self-masks and neighbor-masks. This module employs a Convolutional Neural Network (CNN) to condense the image patches into embedding vectors, capturing spatial information about staining patterns, cell morphology, marker expression levels, and neighborhood context.

#### Language Encoder:

To incorporate semantic understanding of markers and cell types, we employed a language encoder that uses a large language model (LLM) to generate semantically rich embedding vectors in a two-step process (Fig. 2b): First, a frozen LLM explainer is prompted to extract comprehensive knowledge about a marker or cell type, capturing general information, marker-cell type relationships, and alternative names. Next, a frozen LLM embedder converts this semantic information into an information-rich embedding vector. This approach leverages knowledge about the biology of markers and cell types, enabling our model to understand the meaning behind each channel.

#### Channel-wise Transformer:

To allow our model to generalize across marker panels, we use a transformer module with channel-wise attention. This module adds a marker’s image embedding with its corresponding language embedding and applies self-attention to the combined representation (Fig. S2a). Crucially, this self-attention operation is applied across all the markers, similar to how a human might look across the marker images to interpret a stain. While transformers for sequence data typically include a positional encoding, we did not include one. This design choice preserved the length-and-order invariance of self-attention (45) and allowed our model to process inputs from diverse marker panels without modification. We appended a learnable [CLS] token to represent the overall cell and to aggregate information across all channels. The normalized attention weights between this [CLS] token and the marker embeddings in the final layer provide interpretable marker positivity scores. This architecture serves as a fusion point for visual and linguistic information, enabling our model to discern cross-channel correlations while also understanding the biological significance of each marker.

### 2.3 Improved cross-platform generalization through contrastive and adversarial learning

Our training strategy extends the joint vision-language approach from the model architecture to the design of the loss function. Instead of relying on a standard classification loss, we employ the Contrastive Language-Image Pretraining (CLIP) loss, which can effectively integrate image and text information across various tasks (46). We use the same language encoder to embed markers and cell types with corresponding prompts (Table S2). During training, our language-informed vision model was tasked with aligning each cell’s image-marker embedding (i.e., the cell’s [CLS] token embedding taken from the final transformer layer) with the embedding of its corresponding cell type name (Fig. 2a). It was also tasked with minimizing the similarity between the cell’s image-marker embedding with embeddings of incorrect cell type names. This contrastive training approach unifies the visual ([CLS] token embeddings) and textual (cell-type name embeddings) representations in a shared latent space, enhancing the model’s ability to capture nuanced semantic meanings between different cell types. We used a focal-enhanced (47) of the CLIP loss to deal with class imbalance by assigning higher importance to difficult examples. Furthermore, we applied a binary cross-entropy loss to the normalized attention weights to force alignment between the attention weights and marker positivity. For this loss, we used label smoothing (48, 49) to prevent the model from becoming over-confident.

The use of a contrastive loss also enables dynamic inference-time prediction-set binding (Fig. S2d), which allows us to select the target set of cell types during inference, regardless of the cell types the model was trained on. We use this capability to constrain predictions based on tissue types (Fig. S1c), mitigating cross-tissue classification errors. We found this was particularly useful when applying the model to unseen datasets. Furthermore, this approach inherently decouples the training-time and inference-time prediction sets. Therefore, we can train the model with highly granular, tissue-specific subtypes without increasing its susceptibility to classification errors (Table S1). Ultimately, this methodology allows us to enhance prediction specificity without compromising overall model accuracy.

During the development of Expanded TissueNet, we noted significant class imbalances with respect to imaging modalities. To prevent the model from learning representations that are overfit to any single modality, we implemented an auxiliary task that encourages the model to learn invariant representations across imaging platforms. To do so, we added a classification head that takes [CLS] token embeddings and predicts imaging modalities and coupled it with a gradient reversal layer (50). During backpropagation, the gradients were reversed, thus teaching the model to ‘unlearn’ modality-specific differences (Fig. S2c). We found the resulting model developed more robust and general features and focused on the underlying biological characteristics of cells rather than platform-specific features (Fig. 2d, Fig. S4b).

### 2.4 Benchmarking

We sought to evaluate our model’s performance across diverse datasets against other state-of-art approaches. Our analysis encompassed various imaging modalities, tissue types, and cell types, providing a comprehensive view of the model’s effectiveness (Fig. 2e). Fig. 2c showcases example images, visually demonstrating the model’s efficacy across diverse inputs. Additionally, we also benchmarked the marker positivity prediction of our model against Nimbus on both our dataset (Fig. 2f) and Nimbus’s dataset (Fig. S4f). While both models performed optimally on their native datasets and experienced a performance drop on the opposing dataset, our model showed a mitigated decline, underscoring its enhanced generalization.

To assess the modality-invariance of learned features, we applied a two-step dimensionality reduction technique to the cell embeddings: Neighborhood Components Analysis (NCA) (51) followed by t-SNE (52). This approach, previously shown to be effective in revealing latent space structure without overfitting (53), allowed us to visualize the organization of our model’s latent space. The resulting visualization, presented in Fig. 2d, reveals that the embedding space is primarily organized by cell types rather than imaging modalities (Fig. S4b). This organization demonstrates the model’s focus on biologically relevant features over platform-specific characteristics.

We conducted a series of hold-out experiments to evaluate our model’s zero-shot generalization capabilities. In each experiment, we excluded one dataset from training, trained the model on the remaining data, and then tested its performance on the held-out dataset. We benchmarked our model against two baselines: XGBoost (54), a tree-boosting algorithm, and MAPS (20), a neural network-based approach. We note that in this evaluation, differences in marker panels are effectively flagged as missing data for XGBoost and set to zero for MAPS. As shown in Fig. 2g, our model demonstrates favorable performance compared to the baselines across most cell types. This demonstrates that our language-informed vision model effectively leverages semantic understanding to achieve superior generalization across marker panels compared to previous approaches. Moreover, it highlights the value of integrating language into vision information to address the challenges of heterogeneous spatial proteomics data.

## 3 Discussion

This work addresses a critical challenge in spatial proteomics: the need for automated, generalizable, and scalable cell phenotyping tools. Our approach directly tackled the fundamental issue of variability in marker panels across different experiments, enabling learning from multiple data sources. Trained on a diverse dataset, our model demonstrated robust and accurate performance across various experimental conditions, outperforming existing methods in zero-shot generalization tasks.

Our model’s use of raw image data as input represents a significant advancement over traditional cell phenotyping approaches originated from non-spatial single-cell technologies like scRNA-Seq and flow cytometry. These methods rely on mean intensity values extracted from segmented cells and often fall short in coping with the artifacts of spatial proteomic data. Our work adds to the growing body of evidence that image-centric approaches are more robust to segmentation errors, noises, and signal spillovers (18, 26).

A key innovation in our model is the integration of language components to enhance generalization. By incorporating textual information about cell types and markers, we leverage broader biological knowledge that extends beyond the training data. The synergy between visual and linguistic information allows our language-informed vision model to integrate information across a broad set of experimental data, achieving superior performance compared to traditional machine-learning approaches. A key advantage of our approach is its ability to train on multiple datasets simultaneously, leveraging marker correlations across datasets, a capability lacking in existing methods. This unified framework allows continuous model improvement as new data emerges, consolidating the field’s knowledge into a single, increasingly comprehensive model. Given the success of language-informed vision models in handling the heterogeneous marker panels here, applying this methodology to image-based spatial transcriptomics or marker-aware cell segmentation would be a natural extension of this work.

Despite these advances, our method has limitations that merit discussion. First, our zero-shot experiment demonstrates a common characteristic of machine learning systems: while the performance is optimal within the domain of training data, it inevitably degrades when encountering samples too far out-of-domain. Despite our best efforts, Expanded TissueNet does not exhaust all imaging modalities and tissue types. Hence, even though we showed promising improvement compared to existing methods, datasets that substantially diverge from Expanded TissueNet may require additional labeling and model finetuning to achieve adequate performance. We anticipate our labeling software and human-in-the-loop approaches to labeling will enable these efforts and facilitate the collection and labeling of increasingly diverse data, further improving model performance. As demonstrated here and previously (13, 14), integrated data and model development is viable for consortium-scale deep learning in the life sciences. Second, while our work enables generalization across marker panels, generalization across cell types remains an open challenge, given the wide array of tissue-specific cell types. Addressing this limitation is a crucial direction for future investigation. Last, our model is trained on cell patches and cannot access the full image context. While this constraint on the effective receptive field can promote generalization, it may also limit accuracy. Features at longer length scales like functional tissue units and anatomical structures can provide contextual information to further aid cell type determination. Multi-scale language-informed vision models that integrate both local and global features may offer a novel avenue for enhanced performance.

In conclusion, DeepCell Types marks a significant advancement in spatial proteomics data analysis. By directly addressing the challenge of generalization across marker panels, we enabled accurate cell-type labeling for spatial proteomics data generated throughout the cellular imaging community, laying the groundwork for future innovations in cellular image analysis.

## 4 Materials and Methods

### 4.1 Data preprocessing and standardization

We implemented several preprocessing steps that were applied to each marker image independently to ensure consistent and high-quality input for our model. To standardize image resolution, all datasets were resampled to 0.5 microns per pixel (mpp) using nearest-neighbor interpolation and without anti-aliasing. Consequently, 32% of the datasets underwent downsizing, 58% were upsized, and the remaining were unchanged. We thresholded the images using the 99th percentile of all non-zero values per channel per FOV and then applied min-max normalization. Processed images were saved in zarr format, enabling chunked, compressed storage and fast loading. We also standardized marker names and cell type labels across datasets, establishing a canonical name for each marker and cell type, identifying and mapping all alternative names accordingly.

Cell segmentation was performed using the Mesmer algorithm (13). We identified nuclear channels (e.g., DAPI, Histone H3) and cytoplasm/membrane channels (e.g., Pan-Keratin, CD45) to generate accurate whole-cell masks. For each segmented cell, we extracted a 64×64 pixel patch centered on the cell. For cells near image boundaries, we added padding to ensure complete 64×64 patches. To capture segmentation information, we appended a self-mask and a neighbor-mask to each channel’s raw image. This resulted in images of shape (*C*, 3, 64, 64), where *C* is the number of marker images varying by dataset. We zero-padded the tensor to *C*_*max*_ = 75 to allow processing by regular neural networks. A binary padding mask of (*C*_*max*_,) is also generated for later use in the transformer module. Marker positivity labels were generated by manually gating the mean signal intensity for each marker for each dataset.

### 4.2 Model architecture

Our model employs a hybrid architecture, combining Convolutional Neural Network (CNN) and Transformer components to effectively process both image and textual data. Our model architecture has four components:

- *Image encoder*: The image encoder consists of an 11-layer CNN with 2D convolution layers with kernel size 3, padding 1, and interleaved stride size 1 and 2, followed by SiLU activation and batch normalization. We reshape the input tensors from (*B*, *C*_*max*_, 3, 64, 64) to (*B* ∗ *C*_*max*_, 3, 64, 64), allowing a single CNN to process all channels regardless of their marker correspondence. This design ensures the model remains agnostic to specific marker representations. The CNN output is then reshaped into embeddings of size (*B*, *C*_*max*_, 256).
- *Text encoder*: For the text encoder, we used a dense model distilled from the open-source DeepSeek-R1 (55) model built on the Llama-3.3–70B-Instruct model as the LLM expert explainer and its corresponding embedder as the LLM embedder. We prompt the explainer to provide detailed descriptions of each marker type including its general description, alternative names, and cell type correspondence. The resulting 8192-dimensional embeddings are linearly mapped to 256 dimensions to match the image embeddings.
- *Channel-wise transformer*: To merge information contained in the image and text embeddings, we fed them into a transformer module that uses channel-wise attention. A learnable [CLS] token was appended to the embedding tensor to represent the entire cell. The transformer module comprises 5 encoder layers with a hidden dimension of 256 and a feed-forward dimension of 512. We employ a padding mask to exclude dummy channels, ensuring the model focuses only on real channels. This flexible mechanism accommodates varying numbers of input channels.
- *Gradient reversal*: We employed a gradient reversal technique to mitigate systematic bias induced by different imaging modalities. The [CLS] token is fed into a 3-layer MLP classification head for predicting imaging modalities. During backpropagation, we reverse the gradient of the first layer. This approach minimizes the loss on cell type classification while maximizing the loss on imaging modality classification, effectively removing modality-specific information from the learned representations.

To determine marker positivity, the attention weights between the [CLS] token and all other tokens were extracted, normalized to the range [0, 1], and interpreted as marker positivity scores. For cell type classification, we first extracted the [CLS] token embedding from the output layer and reused the text encoder to convert cell type names into embeddings. We trained the [CLS] embedding and cell type name embeddings using the contrastive loss, inspired by CLIP (46). This was achieved by maximizing the cosine similarity between the correct [CLS] embedding and its corresponding cell type name embedding (ground truth similarity of 1), while minimizing it for all other incorrect cell type names (ground truth similarity of 0).

### 4.3 Model training

Our training strategy incorporates three distinct losses: cell type classification, marker positivity prediction, and reverse imaging modality classification. We assign constant weights to the first two components while employing a ramping weight for the third, proportional to the quartic root of the number of epochs. This approach allows the model to initially learn representations best suited for cell type classification and marker positivity prediction before gradually moving towards modality-invariant features to enhance generalization performance.

For cell type classification, we implement a focal version of the contrastive loss (47, 46) with gamma = 2.0. This adaptation addresses class imbalance and improves performance on more challenging categories. The marker positivity prediction utilizes binary cross-entropy loss with label smoothing = 0.2, a technique known to prevent overfitting (48, 49). The reverse imaging modality prediction utilizes cross-entropy loss with label smoothing = 0.01.

**Algorithm 1:** Focal CLIP Contrastive Loss

1:	**procedure** FocalCliPLoss(I,T,γ,τ)	
2:	**Input:** Image embeddings I	
3:	**Input:** Text embeddings T	
4:	**Input:** Focusing parameter γ	
5:	**Input:** Learnable scaling parameter τ	
6:	$I\leftarrow I/\|I{\|}_{2},T\leftarrow T/\|T{\|}_{2}$	▷ Normalize embeddings
7:	$S\leftarrow IT^{⊤}/\tau$	▷ Similarity matrix
8:	$P_{img}\leftarrow softmax(S)$	
9:	$P_{txt}\leftarrow softmax\left(S^{⊤}\right)$	
10:	Y←[0,1,…,N-1]	▷ Groundtruth indices
11:	**procedure** FocalLoss(P,Y)	
12:	ce←NLLLoss(P,Y)	
13:	$P_{t}\leftarrow P[i,Y[i]]$ for all i	▷ Probabilities of all true classes
14:	$\mathrm{loss}\;\leftarrow {\left(1-P_{t}\right)}^{\gamma }\cdot ce$	▷ Focal-weighted loss
15:	**return** mean(loss)	
16:	**end procedure**	
17:	$L_{img}\leftarrow \text{FocalLoss}\left(P_{img}\right)$	
18:	$L_{txt}\leftarrow \text{FocalLoss}\left(P_{txt}\right)$	
19:	$L\leftarrow \left(L_{img}+L_{txt}\right)/2$	▷ Symmetric loss
20:	**return** L	
21:	**end procedure**	

We train the model for 20 epochs using the RAdam optimizer with a learning rate of 10^−4^. We employ several data augmentation techniques to boost generalization, including random flipping, rotation, and image resizing. Further, we randomly drop out 8 marker channels during training to encourage the model to learn robust features that are less dependent on specific markers. We also added random gaussian noises with level = 0.005 to marker name embeddings and cell type embeddings to prevent overfitting and to improve robustness.

### 4.4 Baselines

To benchmark our model’s performance in marker positivity prediction, we compared it to Nimbus (26). Nimbus is a pretrained deep learning model that predicts marker positivity directly, without requiring retraining. We followed the instructions on https://github.com/angelolab/Nimbus-Inference, converting our datasets to TIFF format and executing their preprocessing and prediction protocols.

To benchmark our model’s performance in cell type prediction, we used two established approaches: XGBoost (54) and MAPS (20). We began by extracting features from each dataset and calculating the mean intensity value for each available marker channel in every cell. Next, we compiled a list of all unique markers present across all datasets, which served as a universal reference for data alignment. We then harmonized the data by aligning each dataset to this universal marker list. For markers present in a dataset, we used the calculated mean intensity values, while for absent markers, we inserted a placeholder value (‘NaN‘ for XGBoost, 0 for MAPS).

We implemented XGBoost using the Python XGBoost package (https://github.com/dmlc/xgboost). Placeholder values were explicitly indicated through the data matrix interface. We trained the XGBoost model for 160 epochs using default parameters.

For MAPS, we used the implementation available at https://github.com/mahmoodlab/MAPS. In addition to the marker expression data, we appended a column representing cell size to the input matrix, as per the MAPS protocol. The MAPS model was trained using default parameters for 500 epochs.

## Supplementary Material

Supplement 1
