# Supplementary material for: Generalized cell phenotyping for spatial proteomics with language-informed vision models
Source: bioRxiv. 2025 Aug 22:2024.11.02.621624. Preprint. [Version 3] doi: 10.1101/2024.11.02.621624 (PMC11601246; doi:10.1101/2024.11.02.621624)
Supplement: Supplement 1 [file NIHPP2024.11.02.621624v3-supplement-1.pdf]

bioRxiv preprint doi: https://doi.org/10.1101/2024.11.02.621624; this version posted August 22, 2025. The copyright holder for this preprint (which was not certified by peer review) is the author/funder, who has granted bioRxiv a license to display the preprint in perpetuity. It is made available under aCC-BY 4.0 International license.

Supplementary Information

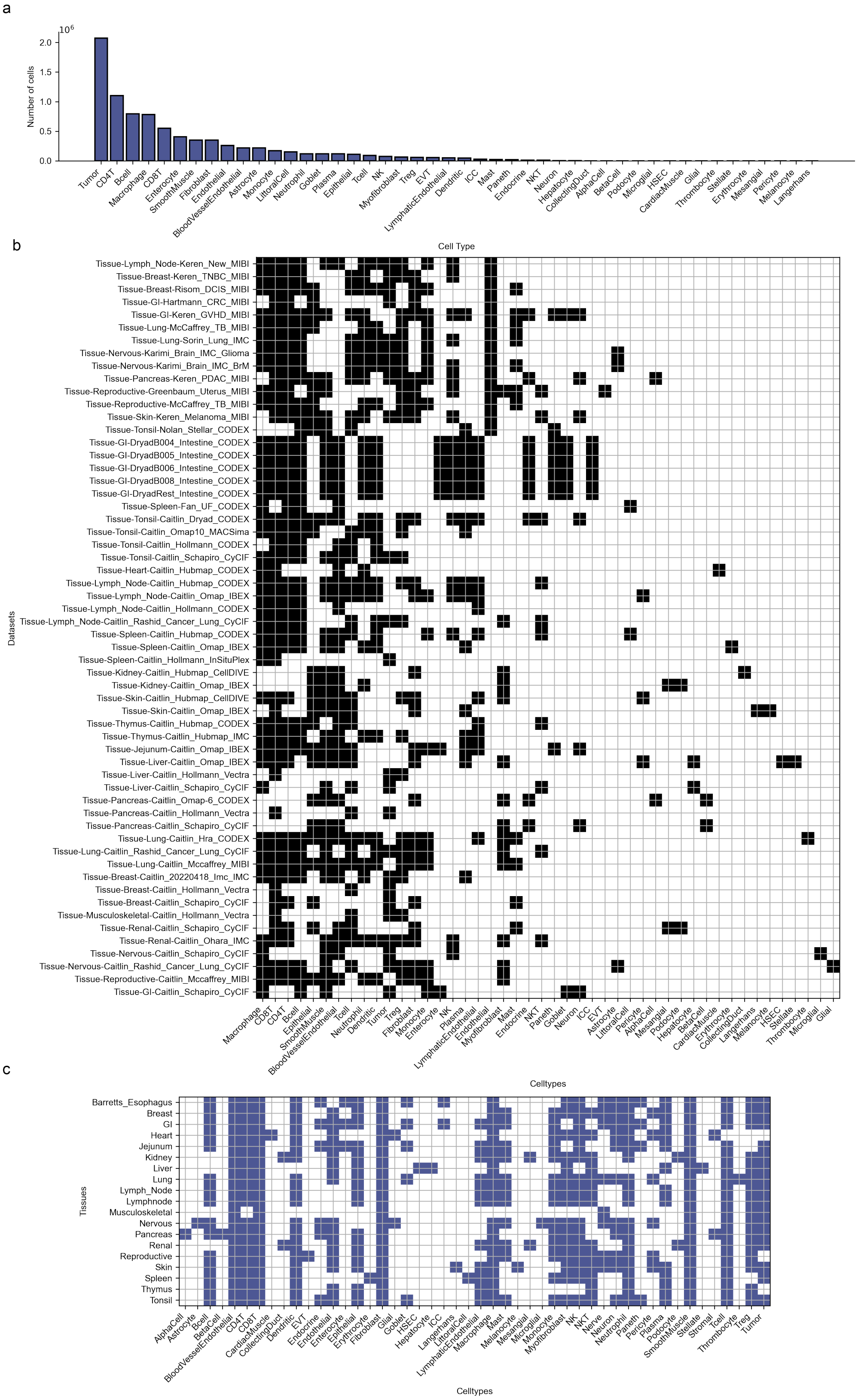

Supplementary Figure S1: **Expanded TissueNet statistics.** a) Distribution of cell type abundances within Expanded TissueNet. b) Heatmap illustrating the presence (dark) or absence (light) of specific cell types across all datasets in Expanded TissueNet. c) Heatmap illustrating the tissue - cell type mapping used for dynamic inference-time prediction-set binding. Dark indicates valid association and light indicates invalid.

bioRxiv preprint doi: https://doi.org/10.1101/2024.11.02.621624; this version posted August 22, 2025. The copyright holder for this preprint (which was not certified by peer review) is the author/funder, who has granted bioRxiv a license to display the preprint in perpetuity. It is made available under aCC-BY 4.0 International license.

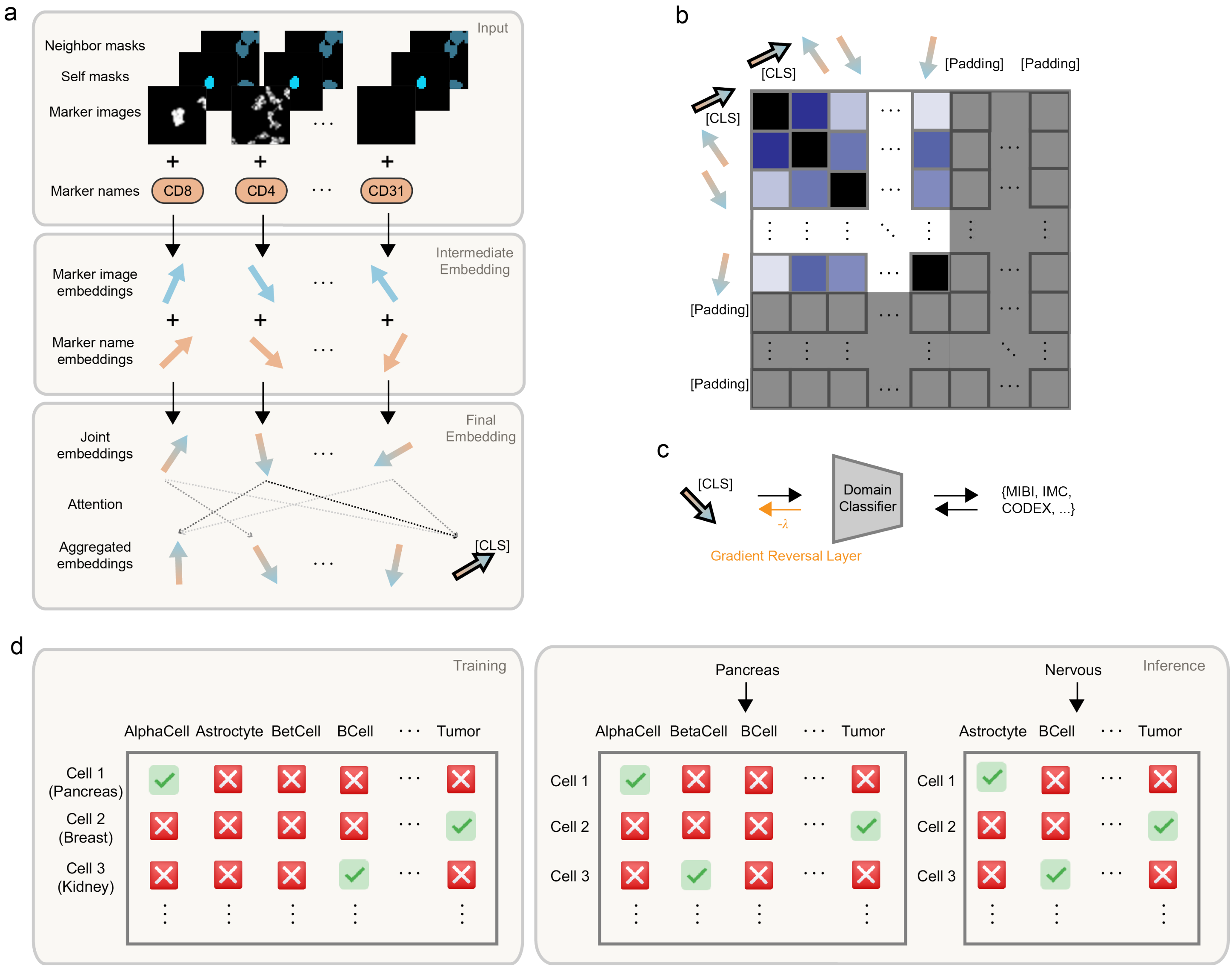

Supplementary Figure S2: **Additional details on model architecture** a) A detailed schematic of the model workflow. Input includes images (raw marker images, and corresponding self masks, and neighbor masks) and text (marker names), which are encoded into embedding separately. Marker image and name embeddings are then added together to form joint embeddings. These serve as tokens for the attention mechanism, modeling inter-channel correlations. A `[CLS]` token that attends to all tokens represents all the information in the images and semantic content of the markers. b) Illustration of attention mechanism. Attention score is calculated across all pairs of embeddings. The binary padding mask is used to mask out invalid channels. We extract the first row as the final attention score used for the marker positivity calculation. c) Domain adversarial training architecture: A Gradient Reversal Layer between the encoder and domain classifier is employed. While the classifier attempts to identify imaging modalities, gradient reversal during backpropagation encourages the encoder to learn modality-invariant features. d) Dynamic inference-time prediction-set binding. During training, we combine samples from different tissues and allow the model to predict from the comprehensive list of cell types; during inference, we choose a subset of cell types based on the tissue type of each dataset in order to reduce errors.

bioRxiv preprint doi: https://doi.org/10.1101/2024.11.02.621624; this version posted August 22, 2025. The copyright holder for this preprint (which was not certified by peer review) is the author/funder, who has granted bioRxiv a license to display the preprint in perpetuity. It is made available under aCC-BY 4.0 International license.

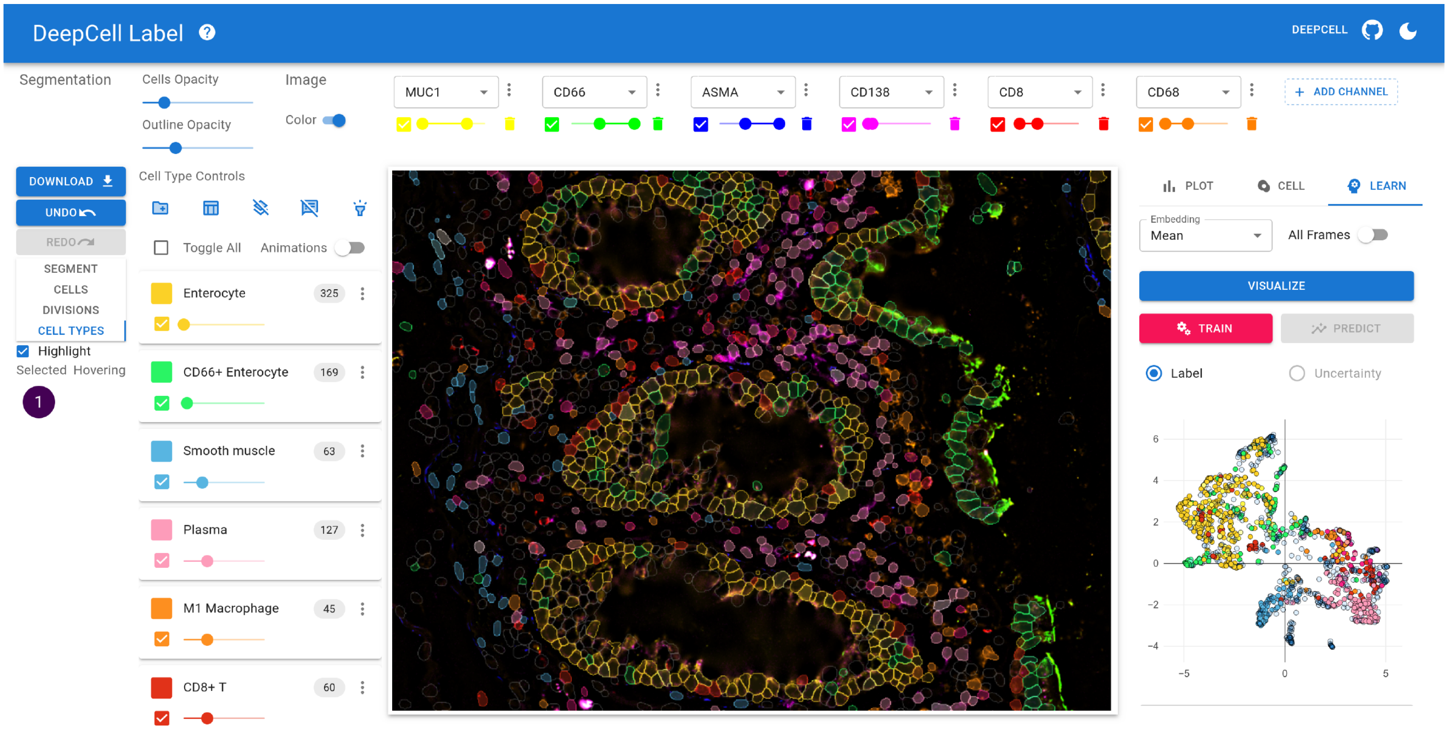

Supplementary Figure S3: **An improved DeepCell Label Interface for spatial proteomic data.** The DeepCell Label interface facilitates expert-in-the-loop development of cell classification models. This browser-native software integrates multichannel visualization, cell-type annotation tools, statistical analysis, and in-browser classifier training based on cell embeddings. With DeepCell Label, we can perform iterative refinement of cell type and marker positivity labels.

bioRxiv preprint doi: https://doi.org/10.1101/2024.11.02.621624; this version posted August 22, 2025. The copyright holder for this preprint (which was not certified by peer review) is the author/funder, who has granted bioRxiv a license to display the preprint in perpetuity. It is made available under aCC-BY 4.0 International license.

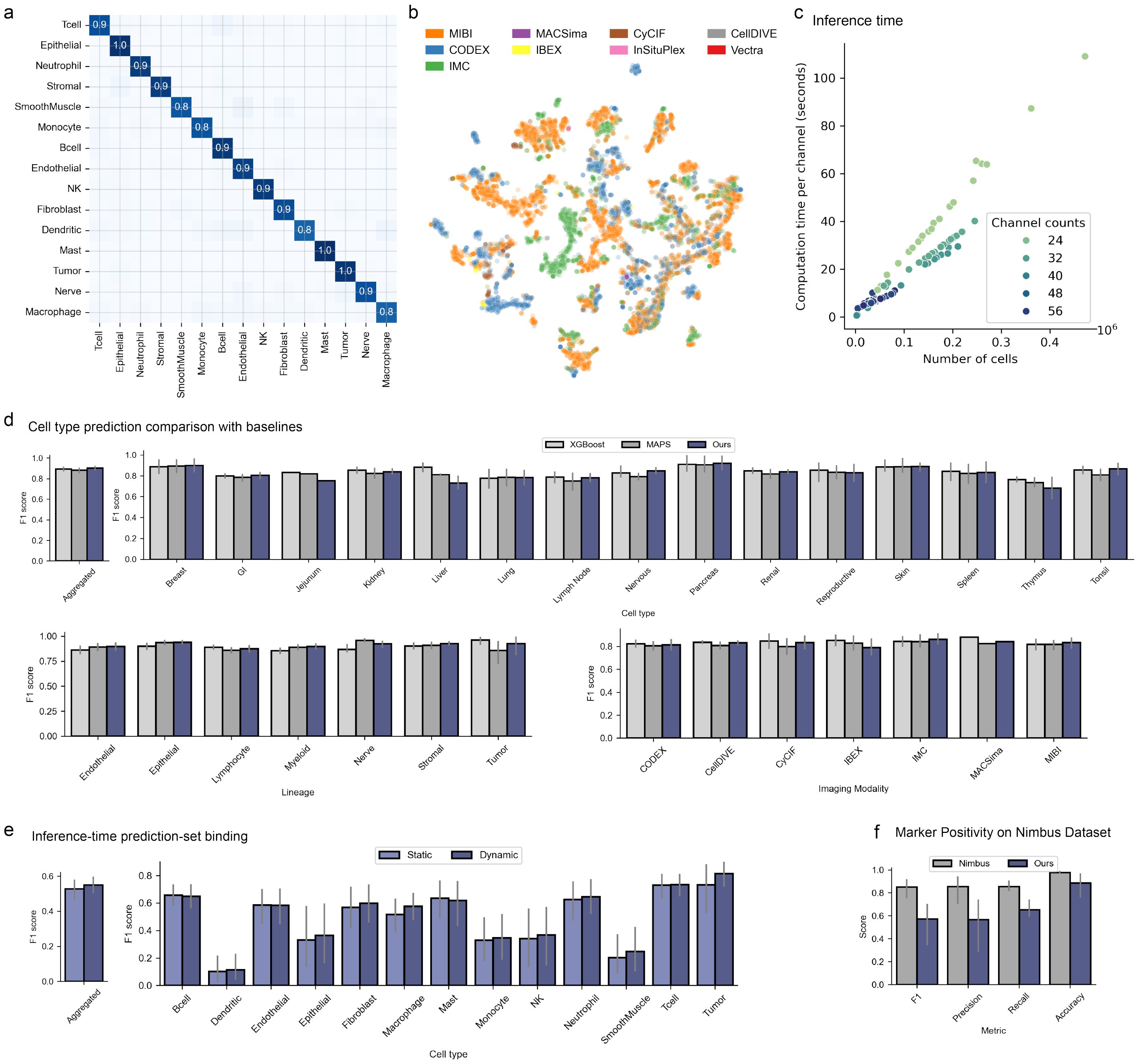

Supplementary Figure S4: **Additional results** a) Confusion matrix for cell type prediction. b) Latent space visualization: an NCA/t-SNE plot of the learned representations, color-coded by imaging modalities. The intermixing of colors demonstrates that the model achieved invariance to imaging modalities. Recoloring the plot by cell type (Fig. 2d) demonstrates that the latent space is organized by cell type instead. c) Per-channel computation time scales linearly with the number of cells. On average, computation time per cell per channel is 0.24 +/- 0.07 milliseconds. d) All methods showed similar performance when evaluated on the in-distribution test set. Aggregated F1 scores are shown on the top left: 0.895 +/- 0.046 (XGBoost), 0.883 +/- 0.061 (MAPS), 0.903 +/- 0.051 (DeepCell Types). We also reported the scored grouped by cell types (top right), lineages (bottom left), imaging modalities (bottom right). e) Inference-time prediction-set binding. Compared to the standard method (static), the dynamic binding of available cell types based on tissue types further improved the scores. Aggregated F1: 0.528 +/- 0.100 (Static), 0.551 +/- 0.08 (Dynamic). f) Comparison of marker positivity prediction on Nimbus dataset.

bioRxiv preprint doi: https://doi.org/10.1101/2024.11.02.621624; this version posted August 22, 2025. The copyright holder for this preprint (which was not certified by peer review) is the author/funder, who has granted bioRxiv a license to display the preprint in perpetuity. It is made available under aCC-BY 4.0 International license.

Our Category	Ontology ID	Ontology Name
Tcell	CL:0000084	T cell
Treg	CL:0000815	regulatory T cell
CD4T	CL:0000624	CD4-positive, alpha-beta T cell
CD8T	CL:0000625	CD8-positive, alpha-beta T cell
NKT	CL:0000814	mature NK T cell
Bcell	CL:0000236	B cell
Plasma	CL:0000786	plasma cell
NK	CL:0000623	natural killer cell
Dendritic	CL:0000451	dendritic cell
Mast	CL:0000097	mast cell
Neutrophil	CL:0000775	neutrophil
Macrophage	CL:0000235	macrophage
Microglial	CL:0000129	microglial cell
Langerhans	CL:0000453	Langerhans cell
Monocyte	CL:0000576	monocyte
Erythrocyte	CL:0000232	erythrocyte
Epithelial	CL:0000066	epithelial cell
CollectingDuct	CL:1001225	kidney collecting duct cell
Goblet	CL:0000160	goblet cell
Paneth	CL:0000510	paneth cell
Enterocyte	CL:0000584	enterocyte
Hepatocyte	CL:0000182	hepatocyte
Podocyte	CL:0000653	podocyte
Endocrine	CL:0000163	endocrine cell
AlphaCell	CL:0000171	pancreatic A cell
BetaCell	CL:0000169	type B pancreatic cell
Endothelial	CL:0000115	endothelial cell
HSEC	CL:1000398	endothelial cell of hepatic sinusoid
LymphaticEndothelial	CL:0002138	endothelial cell of lymphatic vessel
BloodVesselEndothelial	CL:0000071	blood vessel endothelial cell
LittoralCell	CL:1000397	endothelial cell of venous sinus of red pulp of spleen
Stromal	CL:0000499	stromal cell
Fibroblast	CL:0000057	fibroblast
Stellate	CL:0000632	hepatic stellate cell
Myofibroblast	CL:0000186	myofibroblast
SmoothMuscle	CL:0000192	smooth muscle
Pericyte	CL:0000669	pericyte
Mesangial	CL:0000650	mesangial cell
CardiacMuscle	CL:0000746	cardiac muscle cell
ICC	CL:0002088	interstitial cell of Cajal
Nerve	CL:0002319	neural cell
Neuron	CL:0000540	neuron
Glial	CL:0000125	glial cell
Astrocyte	CL:0000127	astrocyte
Tumor	NA	NA
Thrombocyte	CL:0000233	thrombocyte
EVT	CL:0008036	extravillous trophoblast
Melanocyte	CL:0000148	melanocyte

Supplementary Table S1: **Mapping of our cell types to HuBMAP categories based on Cell Ontology (CL).**

bioRxiv preprint doi: https://doi.org/10.1101/2024.11.02.621624; this version posted August 22, 2025. The copyright holder for this preprint (which was not certified by peer review) is the author/funder, who has granted bioRxiv a license to display the preprint in perpetuity. It is made available under aCC-BY 4.0 International license.

Task	Prompt
Marker	You are an expert in spatial proteomics and familiar with protein markers and their cell type associations. Please tell me the functionality, the cell type association and alternative names of marker {marker}. Follow the JSON pattern below: { "[Marker_Type]": { "Functionality": "(Information about functionality of Marker_Type)", "Cell Type Association": "(Information about the cell type associations of Marker_Type)", "Alternative Names": "[(Alternative_Name_1, Alternative_Name_2)]" } }
Cell Type	You are an expert in spatial proteomics and familiar with cell types, their marker associations, and their relations to other cell types. You are most familiar with markers {channel_list} and cell types {celltype_list}. Please tell me the general information, all marker associations, and other related cells of {marker} cell type. Follow the JSON pattern below: { "[Cell_Type]": { "General Information": "[Information about Cell_Type]", "Marker Associations": {"[Marker_1]":"[Information about Marker_1]", "[Marker_2]":"[Information about Marker_2]", "[Marker_3]":"[Information about Marker_3]"}, "Related Cell Types": {"[Related_Cell_Type_1]":"[Information about how Related_Cell_Type_1 relates to Cell_Type]", "[Related_Cell_Type_2]":"[Information about how Related_Cell_Type_2 relates to Cell_Type]"} } }

Supplementary Table S2: **Language Encoder Prompts**
