# Supplementary material for: Generalized cell phenotyping for spatial proteomics with language-informed vision models
Source: bioRxiv. 2024 Nov 17:2024.11.02.621624. Preprint. [Version 2] doi: 10.1101/2024.11.02.621624 (PMC11601246; doi:10.1101/2024.11.02.621624)
Supplement: Supplementary file 1 [file NIHPP2024.11.02.621624v2-supplement-1.pdf]

bioRxiv preprint doi: https://doi.org/10.1101/2024.11.02.621624; this version posted November 17, 2024. The copyright holder for this preprint (which was not certified by peer review) is the author/funder, who has granted bioRxiv a license to display the preprint in perpetuity. It is made available under aCC-BY 4.0 International license.

Supplementary Information

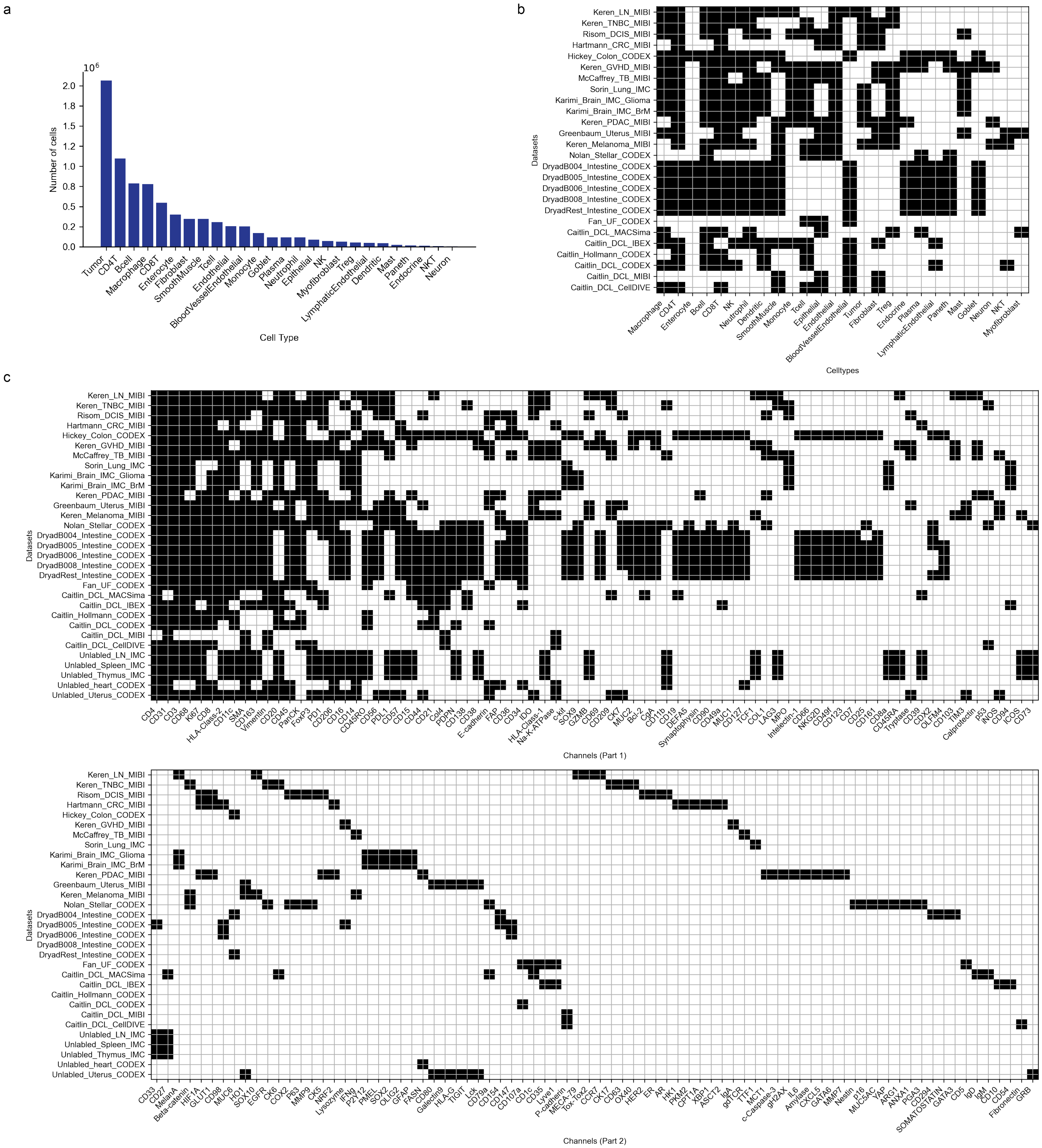

Supplementary Figure S1: **Expanded TissueNet statistics.** a) Distribution of cell type abundances within Expanded TissueNet. b, c) Heatmaps illustrating the presence (dark) or absence (light) of specific cell types (b) and markers (c) across all datasets in Expanded TissueNet.

bioRxiv preprint doi: https://doi.org/10.1101/2024.11.02.621624; this version posted November 17, 2024. The copyright holder for this preprint (which was not certified by peer review) is the author/funder, who has granted bioRxiv a license to display the preprint in perpetuity. It is made available under aCC-BY 4.0 International license.

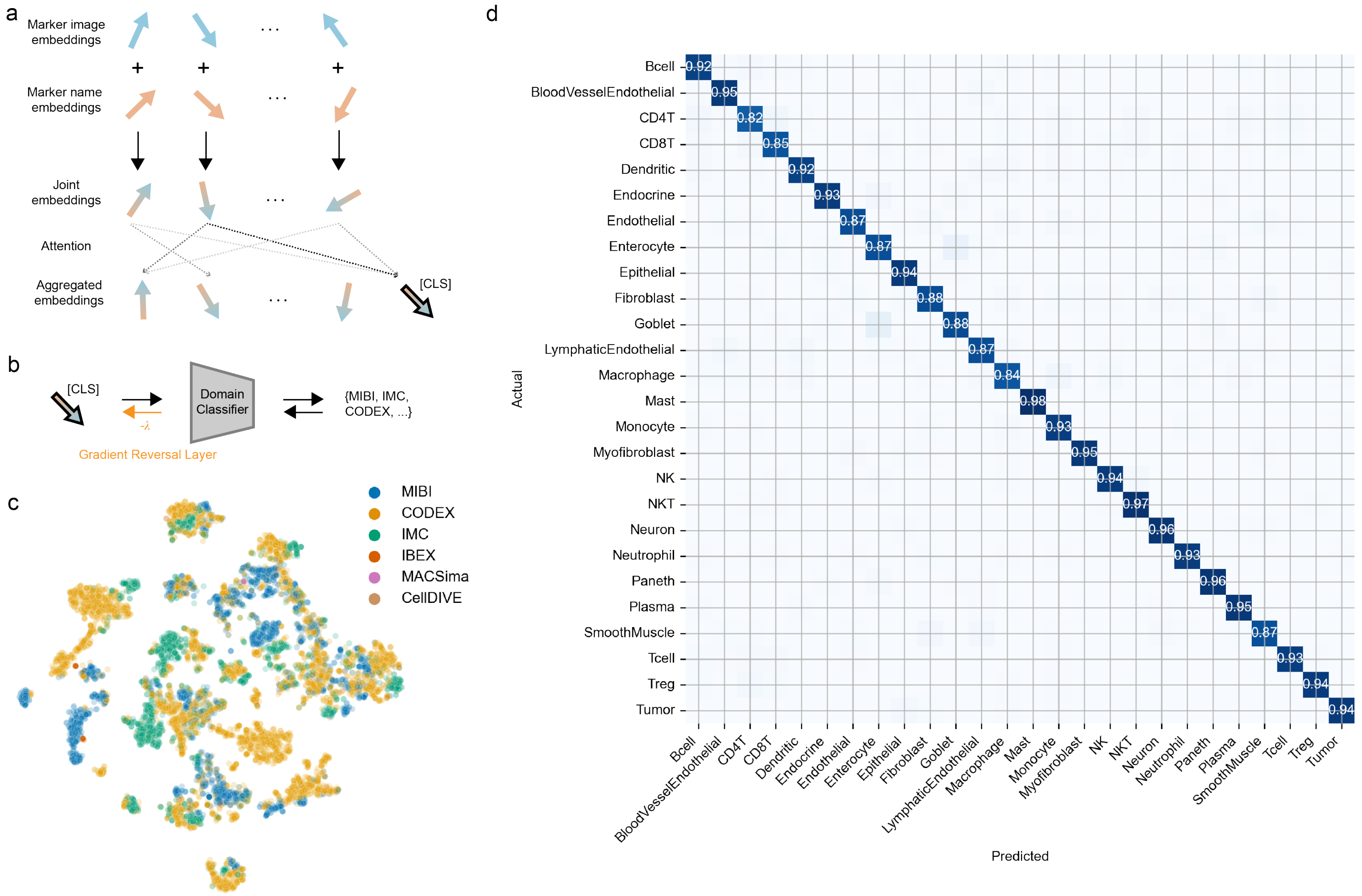

Supplementary Figure S2: **Model architecture and performance.** a) A detailed schematic of the channel-wise attention mechanism. Marker image and name embeddings are added together to form joint embeddings. These serve as tokens for the attention mechanism, modeling inter-channel correlations. A `[CLS]` token that attends to all tokens represents all the information in the images and semantic content of the markers. b) Domain adversarial training architecture: A Gradient Reversal Layer between the encoder and domain classifier is employed. While the classifier attempts to identify imaging modalities, gradient reversal during backpropagation encourages the encoder to learn modality-invariant features. c) Latent space visualization: an NCA/t-SNE plot of the learned representations, color-coded by imaging modalities (MIBI, CODEX, IMC, IBEX, MACSima, CellDIVE). The intermixing of colors demonstrates the model achieved invariance to imaging modalities. Recoloring the plot by cell type (Fig. 2d) demonstrates that the latent space is organized by cell type instead. d) A comprehensive confusion matrix for cell type classification across all cell types.

bioRxiv preprint doi: https://doi.org/10.1101/2024.11.02.621624; this version posted November 17, 2024. The copyright holder for this preprint (which was not certified by peer review) is the author/funder, who has granted bioRxiv a license to display the preprint in perpetuity. It is made available under aCC-BY 4.0 International license.

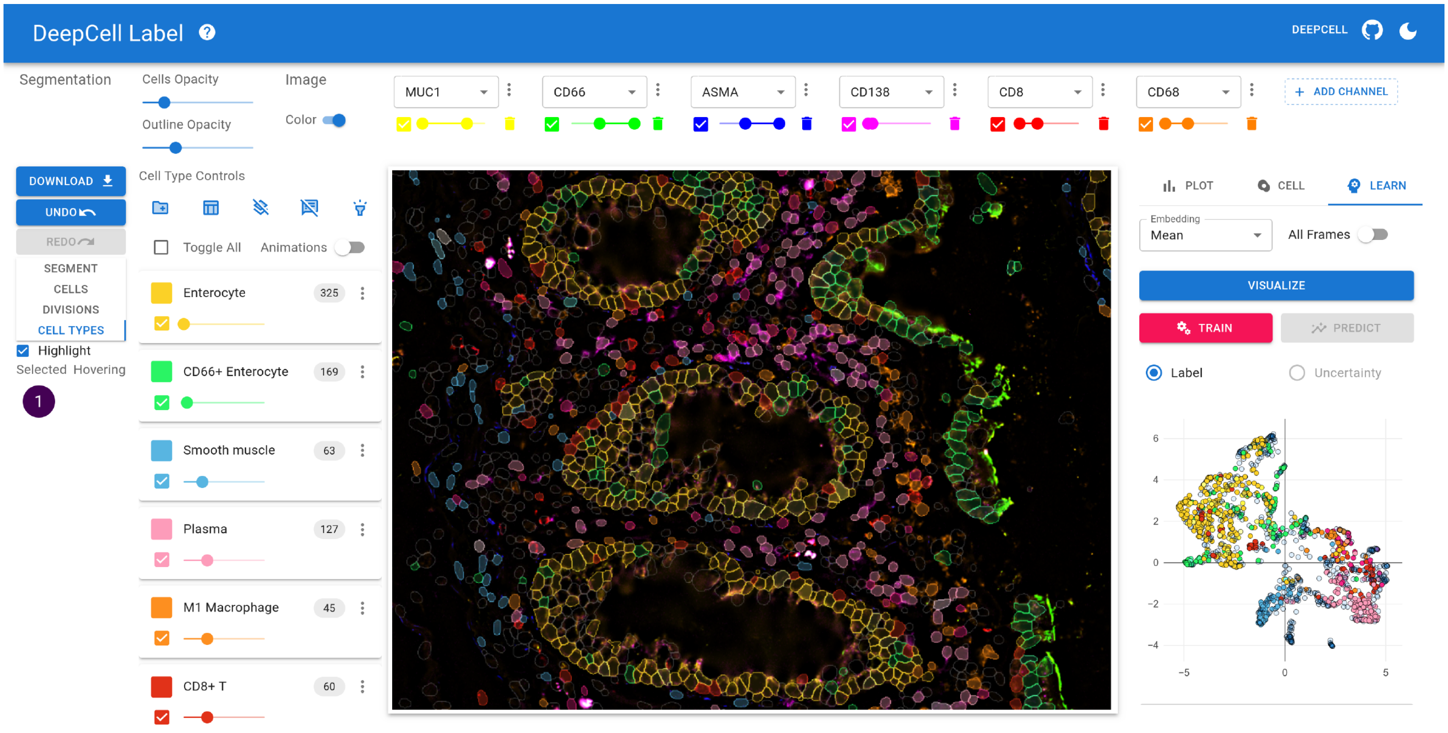

Supplementary Figure S3: **An improved DeepCell Label Interface for spatial proteomic data.** The DeepCell Label interface facilitates expert-in-the-loop development of cell classification models. This browser-native software integrates multichannel visualization, cell-type annotation tools, statistical analysis, and in-browser classifier training based on cell embeddings. With DeepCell Label, we can perform iterative refinement of cell type and marker positivity labels.

bioRxiv preprint doi: https://doi.org/10.1101/2024.11.02.621624; this version posted November 17, 2024. The copyright holder for this preprint (which was not certified by peer review) is the author/funder, who has granted bioRxiv a license to display the preprint in perpetuity. It is made available under aCC-BY 4.0 International license.

Our Category	Ontology ID	Ontology Name
Tcell	CL:0000084	T cell
Treg	CL:0000815	regulatory T cell
CD4T	CL:0000624	CD4-positive, alpha-beta T cell
CD8T	CL:0000625	CD8-positive, alpha-beta T cell
NKT	CL:0000814	mature NK T cell
Bcell	CL:0000236	B cell
Plasma	CL:0000786	plasma cell
NK	CL:0000623	natural killer cell
Dendritic	CL:0000451	dendritic cell
Mast	CL:0000097	mast cell
Neutrophil	CL:0000775	neutrophil
Macrophage	CL:0000235	macrophage
Monocyte	CL:0000576	monocyte
Epithelial	CL:0000066	epithelial cell
Goblet	CL:0000160	goblet cell
Paneth	CL:0000510	paneth cell
Enterocyte	CL:0000584	enterocyte
Endocrine	CL:0000163	endocrine cell
Endothelial	CL:0000115	endothelial cell
LymphaticEndothelial	CL:0002138	endothelial cell of lymphatic vessel
BloodVesselEndothelial	CL:0000071	blood vessel endothelial cell
Fibroblast	CL:0000057	fibroblast
Myofibroblast	CL:0000186	myofibroblast
SmoothMuscle	CL:0000192	smooth muscle
Nerve	CL:0002319	neural cell
Neuron	CL:0000540	neuron
Glia	CL:0000125	glial cell
Tumor	NA	NA

Supplementary Table S1: Mapping of our cell types to HuBMAP categories cased on Cell Ontology (CL).
